# Determinants of variations in sleep patterns across Brazil: Exploring geographic influences

**DOI:** 10.1016/j.sleepx.2024.100137

**Published:** 2024-12-20

**Authors:** Giovana Longo-Silva, Renan Serenini, Roberto Antunes, Márcia Lima, Anny Pedrosa, Risia Menezes

**Affiliations:** a Research Group ‘Chronobiology, Nutrition and Health’ of Federal University of Alagoas, Maceió, Alagoas, Brazil; b Faculty of Economics, Sapienza University of Rome, Rome, Italy; c Institute of Human Sciences, Department of Geography, Federal University of Pelotas, Pelotas, Rio Grande do Sul, Brazil

**Keywords:** Total sleep time, Sleep, Sleep timing, Human geography, Latitude

## Abstract

**Objective:**

To examine the influence of latitude, longitude, sunrise, and daylight, in conjunction with individual and behavioral factors, on sleep duration, wake time, and bedtime in a country with the world's broadest latitude range, yet characterized by homogeneity in language, cultural traits, and consistent time zones.

**Methods:**

Participants (n = 1440; 18-65y) were part of a virtual population-based survey (2021–22). Sleep patterns were spatially represented through maps using Multilevel B-spline Interpolation. Relationships between and within biological/personal/socio-economic, behavioral and environment characteristics, and sleep outcomes were examined by Lasso regression. Restricted cubic splines were employed to examine the shape of the association of latitude and sunrise with sleep variables and of screen time before bed with bedtime.

**Results:**

Latitude emerged as the primary geographic factor influencing variations in sleep duration and wake time, shortening and advancing, respectively, as latitude increased (towards equatorial line). Younger individuals, those living without partners, with depression, engaging in more frequent evening alcohol consumption, and with poorer diet quality, tended to wake up later. All the variables influenced bedtime, with daylight emerging as the environmental factor exerting the strongest association. While the variability in bedtime showed a non-linear association with latitude and sunrise, it displayed a dose-response relationship with screen time before bed.

**Conclusions:**

Given that adults living in the same country, potentially with a similar timetable, could be having shorter sleep durations according to their latitude position, further studies are required to contemplate advocating for policies that implement schedules based on the sun position rather than the national time zone.

## Highlights

• Latitude was the primary influencer of sleep duration and wake time.
• Sleep duration was associated with latitude, longitude, sex, and screen time.
• Latitude, age, marital status, depression, alcohol, and diet influenced wake time.
• Bedtime nonlinearly associated to latitude, but to screen time before bed.

## 1 Introduction

The impact of individual, cultural, and social schedule factors on sleep behaviors has been thoroughly investigated in previous studies [[1], [2], [3]]. Nevertheless, recent research suggests that the environment and geographical features of one's place of residence also contribute to shaping an individual's sleep patterns [4].

Given that light is the strongest ‘zeitgeber’ for all circadian systems [5], variations in light/dark cycles, determined by latitude and longitude in different geographic zones, may interact in diverse ways with the circadian timekeeping system, thereby affecting sleep/wake patterns [[5], [6], [7], [8], [9], [10]].

However, to date, studies examining the relationship between the natural environment and sleep- and circadian-related variables, besides reporting non-consensual results, have been conducted by comparing different countries or by including a few randomly selected cities within the same country [[5], [6], [7], [8], [9], [10]].

In this perspective, Brazil emerges as an optimal setting for investigating potential sleep determinants. With a geographical span extending 39° from its northernmost to southernmost points, encompassing the broadest latitude range in the world, Brazil presents an unparalleled dataset and a unique opportunity to explore diverse geographic and environmental influences on sleep.

Furthermore, Brazil's homogeneity in language, cultural traits, consistent time zones, and absence of daylight-saving time provide an ideal setting to isolate and examine the impact of geography as a primary variable in understanding sleep patterns across the territory. These insights could significantly contribute to mitigating geographic variations in sleep and effectively aiding in the development of public health strategies [11,12].

Therefore, hypothesizing that variations in sleep behaviors could be attributed to environment differences, we examined the influence of latitude, longitude, sunrise, and daylight, in conjunction with biological, personal, socio-economic, and behavioral factors, on sleep duration, wake time, and bedtime.

## 2 Material and methods

### 2.1 Subjects

This study was carried out with data from the SONAR-Brazil Survey, which aims to investigate chronobiological aspects related to sleep, food, and nutrition among Brazilian adults. This research is exploratory in nature, employing a population-based approach with randomized sampling.

Data collection took place exclusively within a virtual environment using a Google Form, from August 2021 to September 2022. The questionnaire comprised four sections: characterization, health, and lifestyle, sleep characteristics, eating and sleeping schedules. The generated responses were automatically stored in spreadsheets compatible with Microsoft Office Excel® and exported to the statistical software Stata® 13 (Stata Corporation) for data analyses.

All data collection procedures have been conducted according to the Declaration of Helsinki and approved by the Committee of Research Ethics.

Participants were adults, non-pregnant, aged between 18 and 65 years, born and residing in all regions of Brazil (n = 2140). After excluding participants who declared being shift workers (n = 90) the final Survey sample comprised 2050 non-pregnant Brazilian adults. Participants with missing data on the city of residence were also excluded from analyses (n = 610). Therefore, our study included 1440 non-pregnant Brazilian adults, from 397 cities across the country.

### 2.2 Sleep traits

Participants were questioned about their sleep habits on a typical weekday (workdays) and weekend (free days) during the last month. The following questions were used to measure usual sleep and wake times: 1. What time do you wake up? 2. What time do you sleep? Responses were provided in local time, in 30-min increments (hh:mm).

Sleep duration (hours) was calculated as the difference between bedtime and wake time. The average weekly sleep duration, wake time, and bedtime were calculated as follows: [(5 × sleep duration/wake time/bedtime on weekdays)+(2 × sleep duration/wake time/bedtime on weekends)]/7^13−15^.

We adopted the “midpoint of sleep on free days corrected for sleep extension on free days (MSFsc)” as an indicator of chronotype, which is proposed to clean the chronotype of the confounder sleep debt. For more details on the methodology see Refs. [15,16].

### 2.3 Biological, personal, and socio-economic characteristics

Participants self-reported their age, sex, education level, marital status, weight, height, and diagnosis of depression. BMI weight(Kg)/height(m) [2] was then calculated.

### 2.4 Behavioral factors

Participants were asked about tobacco use (yes or no) and the frequency (never, sometimes, often, or always) of evening alcohol consumption (after 18:00).

Food consumption was investigated using a food frequency questionnaire comprising 19 food categories, for which participants selected the frequency of weekly consumption: 'never', 'sometimes (1–3 days/week)', 'almost always (4–6 days/week)' or 'always (6–7 days/week)'. Diet quality was evaluated following the methodology proposed in previous studies [[13], [14], [15]].

From the sum of the scores of each food category, the total score of the Diet Quality Index was obtained, which could range from 0 to 57 points, with a higher score suggestive of a higher frequency of consumption of healthier foods and lower frequency of consumption of unhealthy foods. From the available scores, tertiles were created for diet quality classification: 1st tertile–low quality (21–34 points); 2nd tertile–intermediate quality (35–38 points), and 3rd tertile–good quality (39–47 points) [13,14].

The weekly duration of physical activity was calculated (number of days/week x duration/day).

Screen time was assessed through the questions: 1. "In your free time (not counting work/study), how many hours/day do you spend watching TV, on your computer, tablet, or cell phone?". 2. "Right before bedtime, how long do you spend watching TV, on your computer, tablet, or cell phone?”.

Finally, participants were queried about incorporating a pre-bedtime routine of integrative therapies aimed at promoting sleep (meditation, aromatherapy, color therapy, homeopathy, music therapy, flower remedies, yoga).

### 2.5 Geographic parameters

Brazil has a total population of 203,062,512 and is situated along a latitude range from 05°16′north to 33°45′south and a longitude range from 34°47′ to 73°59′ west. Most of the population (92 %) live in the same time zone, concentrated at or near the east coast [17].

The latitude and longitude of each participants’ city were obtained from data base of the Brazilian Institute for Geography and Statistics [17].

The daylight and sunrise data were obtained from a web application <https://sunrise-sunset.org/> and correspond to the exact day on which participants responded to the questionnaire. Hence, these variables reflect variations in solar cues throughout the year of data collection, potentially influencing distinct seasonal sleep patterns.

### 2.6 Statistical analysis

To assess differences in sleep duration, wake time, and bedtime (means and standard deviations, SD) relative to participants' characteristics, Student's t-tests were performed for binary variables, and ANOVA was used for variables with more than two categories. When significant, we explored differences among groups using Bonferroni correction. To compare sleep measures based on geographic location, we categorized the variables of latitude and daylight into quartiles. For latitude, the quartiles were as follows: 1st quartile (<p25): −32.05 to −23.57; 2nd quartile (p25–p50): −23.55 to −21.20; 3rd quartile (p50–p75): −21.19 to −10.74; and 4th quartile (p75–p100): −10.41 to 2.82. For daylight, the quartiles were: 1st quartile (<p25): 10.09 to 10.92; 2nd quartile (p25–p50): 10.92 to 11.45; 3rd quartile (p50–p75): 11.45 to 11.96; and 4th quartile (p75–p100): 11.96 to 12.38.

The distribution of sleep duration, wake time, and bedtime across Brazil was spatially represented through maps generated using the Surface Smoothing Method: Multilevel B-spline Interpolation, through Qgis® 3.34.1 'Prizren' software. This Polynomial Global method falls into the geodeterministic category, where the dataset is spatially generalized and adheres to a polynomial formula for sample points. Conceptually, the global polynomial positions a plane within the sample cloud, and the unknown weight value is subsequently determined based on the value corresponding to the predictive location on that plane. The plane can subsequently be determined for other points in a similar fashion. The Spline method reconstructs the surface by reducing the point cloud to enable the fitting of a weighted cubic B-Spline curve to the adjusted points [18].

Based on the recommended cutoff points for sleep duration [19], maps were generated in four intervals for sleep duration (≤7h, 7.01h–9h, 9.01h–10h and >10h). Bedtime and wake time were categorized based on the 25th and 75th percentiles of its values (bedtime: <23:30, 23:30 to 24:00, >24:00; wake time: <6:18, 6:18 to 7:51, >7:51).

Least Absolute Shrinkage and Selection Operator (Lasso)-regularized general linear models were performed to examine the relationships between and within biological, personal, and socio-economic characteristics (age, sex, education level, marital status, depression, BMI), behavioral factors (alcohol, tobacco, diet quality, physical exercise, screen time, and integrative therapies), environment characteristics (latitude, longitude, sunrise and daylight), and sleep measures (sleep duration, bedtime, wake time).

Lasso is a regularization technique widely employed in statistical modeling, particularly when dealing with high-dimensional data and potential multicollinearity among predictors. Its primary objective is to enhance the accuracy and interpretability of the regression model by introducing a penalty term to the standard linear regression equation. This penalty is based on the sum of the absolute values of the regression coefficients, which encourages sparsity by pushing certain coefficients to exactly zero. In other words, Lasso not only performs variable selection by excluding irrelevant predictors but also effectively reduces the impact of less influential variables, leading to a simpler and more interpretable model.

The inclusion of numerous predictors often leads to multicollinearity issues and increases the risk of overfitting, where the model fits the training data too closely and performs poorly on new, unseen data. Specifically, our dataset includes variables that are known to be highly correlated, as latitude, daylight and sunrise, for instance. Lasso's ability to shrink coefficients, including some to zero, not only aids in identifying the most relevant predictors for predicting the dependent variables but also addresses the potential collinearity among the explanatory variables. This regularization technique facilitates the creation of a parsimonious and robust model, providing a balance between predictive accuracy and model simplicity, which is crucial for understanding and interpreting the factors influencing the sleep-related dependent variables in your analysis.

Restricted cubic splines were employed to examine the shape of the association of latitude and sunrise with sleep outcomes, of screen time before bed with bedtime and of midpoint of sleep with daylight.

Models were adjusted for age, sex, education level, marital status, depression, BMI, alcohol, tobacco use, diet quality, physical exercise, screen time and integrative therapies.

Statistical significance was determined at a threshold of P-value ≤0.05.

## 3 Results

A total of 1440 adults aged 18–65 years participated in the study, with a majority being female (74 %). The average wake time was 7.19 h, and the average bedtime was 23.36 h, resulting in an average sleep duration of 7.82 h.

Table 1 presents the means (±SD) for sleep duration, wake time, and bedtime across various participant characteristics. Significant differences were observed between groups, revealing distinct variations in sleep patterns based on biological, personal, and socio-economic characteristics, as well as behavioral factors and geographic location.

**Table 1:** Sleep duration, wake time, and bedtime by participant characteristics (n = 1,440).

Variables	Total		Sleep Duration^a^ (hour)	Wake time^a^ (decimal time)	Bedtime^a^ (decimal time)
	**n**	**%**	**Means (±SD)**	**Means (±SD)**	**Means (±SD)**
**Total Sample**	1,440	100	7.82 ± 1.16	7.19 ± 1.34	23.36 ± 1.32
**Age (years)**			*p = 0.83*	***p < 0.001***	***p < 0.001***
18-29	585	40.62	7.84 ± 1.16	7.51 ± 1.44^ab^	23.67 ± 1.31^ab^
30-49	661	45.90	7.80 ± 1.13	6.97 ± 1.22^a^	23.16 ± 1.26^a^
50-65	194	13.47	7.84 ± 1.24	6.98 ± 1.26^b^	23.15 ± 1.35^b^
**Sex**			***p < 0.001***	*p = 0.84*	***p < 0.001***
Male	375	26.04	7.54 ± 1.14	7.20 ± 1.41	23.65 ± 1.38
Female	1,065	73.96	7.92 ± 1.15	7.18 ± 1.32	23.26 ± 1.28
**Education level**			*p = 0.52*	***p < 0.001***	***p < 0.001***
Non-Graduated	423	29.38	7.85 ± 1.21	7.44 ± 1.45	23.59 ± 1.37
Graduated	1,017	70.62	7.81 ± 1.13	7.08 ± 1.28	23.27 ± 1.28
**Marital Status**			*p = 0.14*	***p < 0.001***	***p < 0.001***
No stable union	888	61.67	7.79 ± 1.17	7.34 ± 1.40	23.55 ± 1.33
Stable union	552	38.33	7.88 ± 1.14	6.94 ± 1.20	23.07 ± 1.24
**Depression**			*p = 0.60*	***p < 0.001***	***p < 0.001***
Without	1,279	88.82	7.83 ± 1.13	7.14 ± 1.30	23.31 ± 1.28
With	161	11.18	7.78 ± 1.38	7.54 ± 1.59	23.76 ± 1.52
**Overweight (BMI>24.9 kg/m**^**2**^**)**			*p = 0.09*	*p = 0.17*	*p = 0.94*
Without	806	56.01	7.87 ± 1.09	7.23 ± 1.39	23.36 ± 1.32
With	633	43.99	7.77 ± 1.23	7.13 ± 1.28	23.37 ± 1.32
**Alcohol after 18:00**			*p = 0.49*	***p = 0.002***	***p = 0.01***
Never	701	48.68	7.80 ± 1.17	7.08 ± 1.40	23.28 ± 1.35
≥ 1 day/week	739	51.32	7.84 ± 1.15	7.29 ± 1.27	23.45 ± 1.28
**Tobacco smoker**			*p = 0.27*	***p = 0.001***	***p < 0.001***
No	1,341	93.12	7.83 ± 1.14	7.16 ± 1.32	23.32 ± 1.29
Yes	99	6.88	7.70 ± 1.32	7.61 ± 1.53	23.91 ± 1.60
**Diet Quality**			*p = 0.63*	***p < 0.001***	***p < 0.001***
Low (1st tertile)	536	37.22	7.83 ± 1.21	7.43 ± 1.43^ab^	23.60 ± 1.42^ab^
Intermediate (2nd tertile)	509	35.35	7.79 ± 1.19	7.12 ± 1.37^a^	23.34 ± 1.27^ac^
Good (3rd tertile)	395	27.43	7.86 ± 1.04	6.94 ± 1.13^b^	23.08 ± 1.16^bc^
**Physical Exercise**			***p = 0.01***	*p = 0.14*	***p < 0.001***
No	461	32.01	7.71 ± 1.23	7.26 ± 1.41	23.55 ± 1.45
Yes	979	67.99	7.87 ± 1.12	7.15 ± 1.31	23.28 ± 1.24
**Screen time, min/day**			*p = 0.13*	***p < 0.001***	***p < 0.001***
0–119min	277	19.32	7.73 ± 1.11	6.75 ± 1.13	23.02 ± 1.22
≥120min	1,157	80.68	7.84 ± 1.16	7.29 ± 1.37	23.45 ± 1.33
**Screen time before bed, min/day**			*p = 0.36*	***p < 0.001***	***p < 0.001***
0–59min	782	54.31	7.83 ± 1.05	6.97 ± 1.13^ab^	23.14 ± 1.11^ab^
60–119 min	557	38.68	7.79 ± 1.27	7.39 ± 1.52^a^	23.60 ± 1.47^a^
≥120min	101	7.01	7.97 ± 1.26	7.72 ± 1.47^b^	23.76 ± 1.56^b^
**Integrative therapies**			*p = 0.75*	*p = 0.24*	*p = 0.36*
No	1,186	82.36	7.82 ± 1.15	7.17 ± 1.31	23.35 ± 1.31
Yes	254	17.64	7.84 ± 1.20	7.28 ± 1.47	23.43 ± 1.37
**Latitude**^b^			***p < 0.001***	***p < 0.001***	*p = 0.07*
1st quartile	396	27.50	7.98 ± 1.18^a^	7.49 ± 1.38^abc^	23.51 ± 1.31
2nd quartile	326	22.64	7.86 ± 1.14^b^	7.19 ± 1.20^ad^	23.33 ± 1.22
3rd quartile	358	24.86	7.86 ± 1.08^c^	7.13 ± 1.28^b^	23.27 ± 1.32
4th quartile	360	25.00	7.58 ± 1.18^abc^	6.91 ± 1.42^cd^	23.33 ± 1.40
**Daylight**^c^			***p = 0.003***	***p < 0.001***	***p < 0.001***
1st quartile	360	25.00	7.94 ± 1.17^a^	7.32 ± 1.22^a^	23.38 1.19
2nd quartile	360	25.00	7.92 ± 1.15^b^	7.18 ± 1.34^b^	23.26 1.37^a^
3rd quartile	361	25.07	7.68 ± 1.13^ab^	6.90 ± 1.33^abc^	23.22 1.34^b^
4th quartile	359	24.93	7.78 ± 1.15	7.35 ± 1.43^c^	23.59 1.33^ab^

Abbreviation: BMI, Body Mass Index.

P-values are derived from Student's t-tests for binary variables and ANOVA for variables with more than two categories. When significant, differences among groups were explored using Bonferroni correction. Superscript letters (a, b, c) indicate significant differences after Bonferroni correction.

Significant associations (P-values <0.05) are highlighted in bold.

a Weekly Average: [((Weekday value*5) + (Weekend value*2))/7].

b Minimum and Maximum values by Quartiles of Latitude: 1st quartile (<P25): -32.05 to −23.57; 2nd quartile (P25–P50): -23.55 to −21.20; 3rd quartile (P50–P75): -21.19 to −10.74; 4th quartile (P75–P100): -10.41 to 2.82.

c Minimum and Maximum values by Quartiles of Daylight: 1st quartile (<P25): 10.09 to 10.92; 2nd quartile (P25–P50): 10.92 to 11.45; 3rd quartile (P50–P75): 11.45 to 11.96; 4th quartile (P75–P100): 11.96 to 12.38.

When comparing age groups, participants aged 18–29 years exhibited the latest wake and bedtimes in comparison to older groups (both p < 0.001). On average, women went to bed earlier (23.26 vs. 23.65, p < 0.001) and slept longer (7.92 vs. 7.54 h, p < 0.001) than men, though no statistically significant difference was found in wake time between sexes.

Participants with higher education, those in stable unions, those without depression, non-smokers, and individuals who did not consume alcohol in the evening all woke up and went to bed earlier than their counterparts (all p ≤ 0.01). However, no significant differences in sleep duration were observed within these groups.

In terms of behavioral factors, individuals with poorer diet quality tended to wake up and go to bed later than those with intermediate or high diet quality. Participants who engaged in physical exercise slept longer and went to bed earlier compared to those who were sedentary (p ≤ 0.01). Furthermore, participants reporting less than 2 h of screen time during leisure and less than 1 h of screen exposure immediately before bed also woke up and went to bed earlier (all p < 0.001).

With respect to geographic variables, analysis by latitude and daylight quartiles revealed distinct patterns. Those residing at higher latitudes (4th quartile) had shorter sleep durations compared to all other quartiles, whereas individuals living at lower latitudes (1st quartile) woke up later than participants in other quartiles. Bedtimes did not differ significantly across latitude quartiles.

In terms of daylight exposure, participants living in areas with the shortest daylight duration (1st quartile) reported the longest sleep durations. The earliest wake times were observed in the 3rd daylight quartile, while participants residing in locations with the longest daylight duration (4th quartile) had the latest bedtimes (all p < 0.01). It is important to note that daylight refers to the specific length of sunlight in each participant's city on the day they completed the questionnaire.

**Fig. 1:**
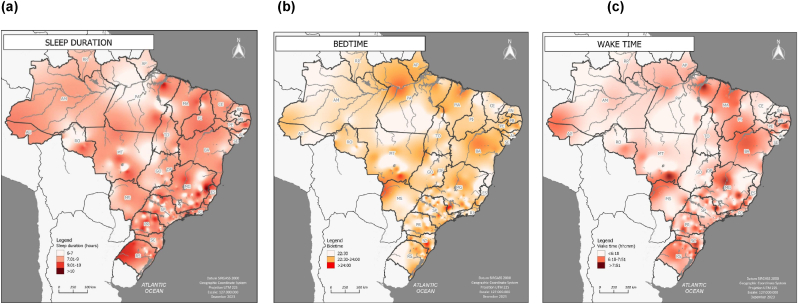
Spatial representation of sleep duration (a), bedtime (b), and wake time of participants (n = 1440).

These geographic variations are further confirmed in Fig. 1, which provides a spatial representation of participants based on their sleep duration, bedtime, and wake time. In cities located at lower latitudes (farther from the equator), particularly in the southeast and south regions of Brazil, sleep duration (Fig. 1a) is longer compared to those at medium to low latitudes.

It is also observed that residents of cities situated at higher latitudes (closer to the equator, where sunrise/sunset are earlier) tend to sleep and wake up earlier (Fig. 1b and c). The concentration of points representing individuals who go to bed later (>24:00), is predominantly located in the central-west, southeast, and southern regions of Brazil, aligning with medium to low latitudes (Fig. 1b).

Lasso regression models (Table 2) provided interesting insights. Longitude, latitude, sex, and screen time were the only variables deemed significant in relation to sleep duration. The nonzero coefficients associated with these variables suggest that they have a meaningful impact on sleep duration, while the coefficients for other predictors are effectively shrunk to zero. The negative coefficient for longitude (−0.017) suggests that as the longitude of the city increases, sleep duration tends to decrease slightly. The negative coefficient for latitude (−0.109) confirm that higher latitudes are associated with a reduction in sleep duration. The positive coefficient for sex (0.143) indicates that being male is associated with a slight increase in sleep duration. The coefficient for screen time (0.040) suggests that an increase in screen time is associated with a corresponding increase in sleep duration.

**Table 2:** Lasso regression analysis for biological, personal, and socio-economic traits, behavioral factors, and geographic characteristics predicting sleep duration, wake time, and bedtime (n = 1440).

	Variables	Standardized Coefficients		
		Sleep Duration	Wake time	Bedtime
**Biological, personal, and socio-economic characteristics**	**Age (years)**	–	−0.133	−0.09
	**Sex (Male)**	0.143	–	−0.183
	**Education level**	–	–	−0.017
	**Marital Status (stable union)**	–	−0.055	−0.155
	**Depression (yes)**	–	0.089	0.138
	**BMI**	–	–	0.014
**Behavioral factors**	**Alcohol (day/week)**	–	0.034	0.033
	**Tobacco (yes)**	–	–	0.04
	**Diet Quality (score)**	–	−0.085	−0.121
	**Physical Exercise (day/week)**	–	–	−0.017
	**Screen time (minutes)**	0.040	0.205	0.131
	**Integrative therapies (yes)**	–	–	0.022
**Geographic Location**	**Longitude**	−0.017	–	0.016
	**Latitude**	−0.109	−0.159	−0.079
	**Daylight (hour)**	–	–	0.159
	**Sunrise (hh:mm)**	–	–	–

Abbreviation: BMI, Body Mass Index.

- coefficient is zero in multiple Lasso regression analysis.

Regarding wake time, the city latitude has a negative impact, with a coefficient of −0.159, suggesting that for each standard deviation increased in latitude, the wake time decreases by approximately 10 min, i.e., people living in the southern part of the country, are more likely to wake up later, compared to people living in the northern part (Table 2).

To illustrate the result, in our dataset the standard deviation of the latitude is around 7°, meaning that a change from the southernmost state to the northernmost state implies in a decrease of waking time of almost 1 h, keeping fixed the other factors. The result is very similar to the previous one, the influence of latitude in the sleep duration, providing more evidence in favor of the effects of this variable. However, the effect is considerably more intense here. The age coefficient indicates that, on average, the increase of a standard deviation in age is associated with a 0.133-h decrease in wake time. Marital status, represented by the coefficient of −0.056, suggests that being married/living with partner is associated with an advance in wake time. The diagnosis of depression was associated with a delay in wake time by 0.091 h. More frequent evening alcohol intake was associated with a slight delay in wake time (0.034). The diet quality, reflected in the coefficient of −0.085, suggests that one-unit improvement in diet quality score is linked to a decrease in wake time by approximately 5 min (Table 2).

Finally, age and marital status were found to be negatively related to bedtime, with coefficients of −0.09 and −0.155, respectively, suggesting that older individuals and those who are married/living with partner tend to go to bed earlier. Additionally, depression showed a positive coefficient of 0.138, implying a potential delay in bedtime among individuals with depression. Other noteworthy factors influencing bedtime included screen time, with a positive coefficient of 0.131, and daylight duration with a positive coefficient of 0.159. Latitude and longitude are suggested to influence bedtime, albeit to a lesser extent (Table 2).

**Fig. 2:**
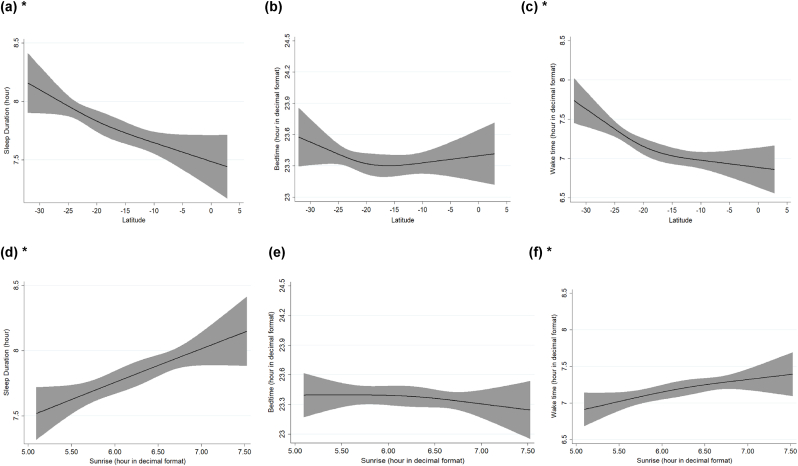
Restricted Cubic Spline Regression represents the association of**latitude and sunrise with sleep duration, bedtime, and wake time** among Brazilian adults (n = 1440)**.** Models are adjusted for age, sex, education level, marital status, depression, BMI, alcohol, tobacco, diet quality, physical exercise, screen time and integrative therapies. Black lines plot the predicted sleep values with 95 % confidence intervals (grey fill). ∗*Significant associations (P-values* ≤ *0.05)*.

**Fig. 3:**
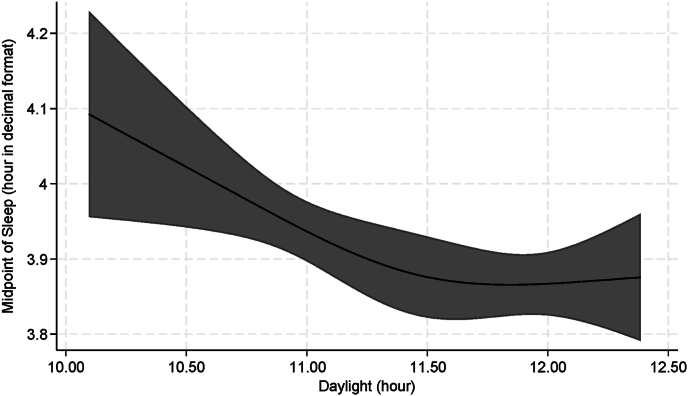
Restricted Cubic Spline Regression represents the association of**daylight with midpoint of sleep** among Brazilian adults (n = 1440)**.** Model is adjusted for age, sex, education level, marital status, depression, BMI, alcohol, tobacco, diet quality, physical exercise, screen time and integrative therapies. Black line plot the predicted midpoint of sleep values with 95 % confidence intervals (grey fill). ∗*Significant association (P-value* < *0.05)*.

**Fig. 4:**
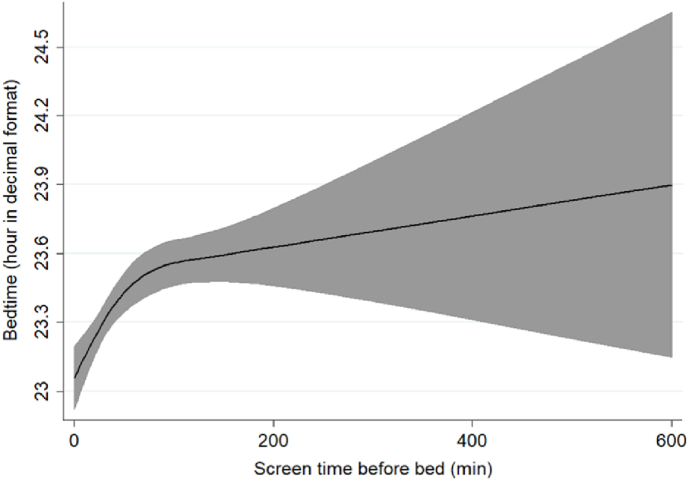
Restricted Cubic Spline Regression represents the association of**screen time use before bed with bedtime** among Brazilian adults (n = 1440)**.** Models are adjusted for age, sex, education level, marital status, depression, BMI, alcohol, tobacco, diet quality, physical exercise, integrative therapies, latitude, longitude and sunrise. Black lines plot the predicted bedtime values with 95 % confidence intervals (grey fill). ∗ Significant association (P-value <0.001).

Restricted cubic splines depicts similar results, illustrating that as latitude increases towards the northern regions, sleep duration decreases (Fig. 2a), and wake time advances (Fig. 2c). Furthermore, with a delayed sunrise, sleep duration (Fig. 2d) increases, and wake time (Fig. 2f) also delays. Fig. 3 illustrates a negative association between sleep midpoint and daylight duration: as the length of daylight increases, the sleep midpoint shifts earlier. While notably, the variability in bedtime exhibited a non-linear association with latitude (Fig. 2b) and sunrise (Fig. 2e), the use of electronic devices with illuminated screens before bed was linearly associated with bedtime (Fig. 4).

## 4 Discussion

Our study analyzed data from a large sample of adult general population, sharing a constant time zone throughout the year, and a potentially similar culture and timetable, along all latitudinal clines of the Brazilian territory. Conscious that biological, personal, socio-economic, and behavioral determinants cannot be ruled out [4], we aimed to test the hypothesis that geographic characteristics also play a role in in sleep patterns variations.

As hypothesized, environmental characteristics were associated with sleep behaviors, with latitude identified as the primary geographic factor explaining variations of sleep duration and wake time across the country. As latitude increases (moving towards the northern/equatorial line), wake time advances, and sleep duration shortens. Additionally, consistent with previous studies, a positive dose-response relationship was observed between sunrise and both wake time and sleep duration, while a negative dose-response association was found between sleep midpoint and daylight duration [5,20,21].

Through Lasso regression, we found that sleep duration was associated with not only latitude and longitude, but also with sex and screen time. Interestingly, an increase in screen time implicated in an extended duration of sleep. While there is an indisputable negative impact of illuminated screen electronic devices on sleep quality, it's essential to note that this variable encompasses the overall daily screen exposure (assessed as a proxy of sedentary behavior) [22,23]. The literature has already reported associations between sedentary behavior in screen time with both 'short' and 'long' sleep durations [24].

Wake time was also associated with biological, personal, socioeconomic, and behavioral factors, indicating a tendency for earlier wake-up times across the lifespan. Individuals living without partners, with depression, engaging in more frequent evening alcohol consumption, and having lower diet quality scores tended to wake up later. These results agree with the literature [4].

Bedtime was the sleep variable most influenced by multiple factors. Daylight emerged as the most significant environmental factor, while latitude and longitude are suggested to influence bedtime to a lesser extent.

Furthermore, all other biological, personal, socio-economic, and behavioral variables demonstrated an effect on bedtime. Importantly, we found a linear positive association between screen time right before bed and bedtime.

From this, we propose that environmental characteristics and solar cues indeed impact sleep patterns but are being attenuated and/or ignored in the modern society, particularly around bedtime.

In the relatively recent past, the availability of artificial light has substantially changed the light environment, particularly during evening and nighttime [25]. While the precise impact of artificial light availability remains unclear, evening light exposure has been demonstrated to suppress melatonin, alter circadian rhythms, and disrupt sleep in humans, contributing to delayed bedtimes and worsening sleep quality [22,[26], [27], [28], [29]].

It is noteworthy that, even if not directly contributing to short sleep duration, a later bedtime may lead to circadian disruption and an increased opportunity for late-night eating-occurring during the biological night relative to melatonin onset-potentially acting as a risk factor for sleep disturbance and metabolic-related outcomes [15,30,31].

### 4.1 Limitations

Our study has a few limitations, starting with the use of self-reported questionnaires which are prone to underreporting or misreporting. However, precise questions were used to investigate sleep domains, the questionnaire specified that responses should be based on recent behaviors (last month) and, to guarantee data as close as possible to the real usual behavior, the questionnaire differentiated weekdays (work days) and weekends (free days). In addition, we recognize that a general weakness of cross-sectional studies is that the direction of the relationship, and possible pathways of causation, can only be hypothesized.

However, our study has several notable strengths. A key advantage is the use of a large sample of Brazilian adults, which enhances the external validity of our findings. Additionally, Brazil's unique geographic and cultural context—without time zone changes or daylight saving time—provided an opportunity to analyze sleep patterns without the interference of these factors, allowing for a clearer assessment of the impact of environmental variables such as latitude, longitude, and sunlight exposure. Furthermore, the use of geographic data corresponding to the exact day the questionnaire was completed enabled precise measurement of seasonal variations in sleep habits. Another strength of the study was the application of advanced statistical techniques, such as Lasso regression, which enabled the identification and control of a broad range of behavioral, lifestyle, and personal characteristics, in addition to geographic variables. This approach facilitated a more comprehensive analysis of the interactions between environmental factors and modern lifestyle.

## 5 Conclusions

Our findings suggest that populations living at higher latitudes tend to wake up earlier and experience shorter sleep durations. However, the minimal influence of natural environmental factors on bedtime suggests a growing disconnection between modern societal routines and the natural light-dark cycle. This disconnect highlights the urgent need for a stronger focus on sleep and circadian hygiene, particularly by avoiding the use of electronic devices, especially during the biological night. Emphasizing this in clinical practice and health promotion strategies is crucial to improve sleep quality and mitigate the negative effects of short sleep duration and circadian misalignments.

Although individuals within the same country often follow similar schedules, those living at different latitudes may still experience significant variations in sleep duration. This highlights the importance of policy reforms that account for solar positioning rather than rigidly adhering to national time zones. Aligning time zones with local solar time could help reduce circadian misalignment, a well-documented factor that negatively impacts sleep duration and quality. Additionally, policies that promote later or more flexible work and study hours could further support these adjustments, facilitating better alignment with individual circadian rhythms and accommodating seasonal variations in sunlight. This would create a biologically appropriate framework for daily activities and social routines.

## CRediT authorship contribution statement

**Giovana Longo-Silva:** Writing – original draft, Visualization, Validation, Supervision, Project administration, Methodology, Investigation, Funding acquisition, Formal analysis, Data curation, Conceptualization. **Renan Serenini:** Writing – original draft, Visualization, Validation, Methodology, Investigation, Formal analysis, Data curation. **Roberto Antunes:** Writing – original draft, Formal analysis. **Márcia Lima:** Writing – review & editing, Methodology, Investigation, Data curation, Conceptualization. **Anny Pedrosa:** Writing – review & editing, Methodology, Investigation, Data curation. **Risia Menezes:** Writing – review & editing.

## Ethical committee permission

All procedures performed in this study, involving human participants, were in accordance with the ethical standards of the institutional and/or national research committee and with the 1964 Helsinki Declaration and its later amendments or comparable ethical standards. SONAR-Brazil Survey*(‘SONAR: investigações cronobiológicas do sono*, *alimentação e nutrição’*) has been approved by the Committee of Research Ethics of the Federal University of Alagoas (CAAE: 48689221.3.0000.5013).

## Data visualization

Data described in the manuscript will be made available upon request pending application and approval.

## Declaration of generative AI in scientific writing

The authors declare that Generative AI and AI-assisted technologies were not utilized in the writing process.

## Funding

This work was supported by ‘Fundação de Amparo à Pesquisa do Estado de Alagoas - FAPEAL’ (Grant/Award Number: 60030.0000002539/2022). AP received a master's scholarship from FAPEAL and ML received a master's scholarship from ‘Coordenação de Aperfeiçoamento de Pessoal de Nível Superior – CAPES’.

## Declaration of competing interest

The authors declare that they have no known competing financial interests or personal relationships that could have appeared to influence the work reported in this paper.

## References

[1] Grandner M.A., Jackson N.J., Izci-Balserak B. (2015). Social and behavioral determinants of perceived insufficient sleep. Front Neurol.

[2] Skeldon A.C., Derks G., Dijk D.J. (2015). Modelling changes in sleep timing and duration across the lifespan: changes in circadian rhythmicity or sleep homeostasis?. Sleep Med Rev.

[3] Skeldon A.C., Dijk D.J., Derks G. (2014). Mathematical models for sleep-wake dynamics: comparison of the two-process model and a mutual inhibition neuronal model. PLoS One.

[4] Philippens N., Janssen E., Kremers S., Crutzen R. (2022). Determinants of natural adult sleep: an umbrella review. PLoS One.

[5] Roenneberg T., Merrow M. (2007). Entrainment of the human circadian clock. Cold Spring Harbor Symp Quant Biol.

[6] Benedito-Silva A.A., Menna-Barreto L., Alam M.F. (1998). Latitude and social habits as determinants of the distribution of morning and evening types in Brazil. Biol Rhythm Res.

[7] Brockmann P.E., Gozal D., Villarroel L. (2017). Geographic latitude and sleep duration: a population-based survey from the Tropic of Capricorn to the Antarctic Circle. Chronobiol Int.

[8] Friborg O., Bjorvatn B., Amponsah B., Pallesen S. (2012). Associations between seasonal variations in day length (photoperiod), sleep timing, sleep quality and mood: a comparison between Ghana (5°) and Norway (69°): seasonal variations in sleep patterns. J Sleep Res.

[9] Leocadio-Miguel M.A., Louzada F.M., Duarte L.L. (2017). Latitudinal cline of chronotype. Sci Rep.

[10] Grandner M.A., Smith T.E., Jackson N. (2015). Geographic distribution of insufficient sleep across the United States: a county-level hotspot analysis. Sleep Health.

[11] Fang S.C., Subramanian S.V., Piccolo R. (2015). Geographic variations in sleep duration: a multilevel analysis from the Boston Area Community Health (BACH) Survey. J Epidemiol Community Health.

[12] Grandner M.A., Jackson N.J., Pigeon W.R., Gooneratne N.S., Patel N.P. (2012). State and regional prevalence of sleep disturbance and daytime fatigue. J Clin Sleep Med.

[13] Longo-Silva G., de Oliveira P.M.B., Pedrosa A.K.P. (2022). Breakfast skipping and timing of lunch and dinner: relationship with BMI and obesity. Obes Res Clin Pract.

[14] Longo-Silva G., Pedrosa A.K.P., de Oliveira P.M.B. (2023). Beyond sleep duration: sleep timing is associated with BMI among Brazilian adults. Sleep Med X.

[15] Pedrosa A.K.P., Lima M.O., Oliveira P.M.B. (2023). Circadian dinner timing and BMI among adults in a Brazilian national survey. Obes Med.

[16] Roenneberg T., Pilz L.K., Zerbibi G., Winnebeck E. (2019). Chronotype and Social Jetlag – a [self] critical review. Biology.

[17] IBGE. Instituto Brasileiro de Geografia e Estatística (2022). Population census. https://www.ibge.gov.br/en/statistics/social/labor/22836-2022-census-3.html.

[18] Rouhani M., Sappa A.D., Boyer E. (2015). Implicit B-spline surface reconstruction. IEEE Trans Image Process.

[19] Ohayon M., Wickwire E.M., Hirshkowitz M. (2017). National Sleep Foundation's sleep quality recommendations: first repost. Sleep Health.

[20] Shawa N., Roden L.C. (2016). Chronotype of South African adults is affected by solar entrainment. Chronobiol Int.

[21] Walch O.J., Cochran A., Forger D.B. (2016). A global quantification of “normal” sleep schedules using smartphone data. Sci Adv.

[22] Castro-Santos L., Lima M.O., Pedrosa A.K.P. (2023). Sleep and circadian hygiene practices association with sleep quality among Brazilian adults. Sleep Med X.

[23] Yang Y., Shin J.C., Li D., An R. (2017). Sedentary behavior and sleep problems: a systematic review and meta-analysis. Int J Behav Med.

[24] Štefan L., Horvatin M., Baić M. (2019). Are sedentary behaviors associated with sleep duration? A cross-sectional case from Croatia. Int J Environ Res Public Health.

[25] Barentine J. (2023). Artificial light at night: state of the sciense 2023. Zenodo.

[26] Cho Y., Ryu S.H., Lee B.R. (2015). Effects of artificial light at night on human health: a literature review of observational and experimental studies applied to exposure assessment. Chronobiol Int.

[27] Duffy J.F., Czeisler C.A. (2009). Effect of light on human circadian physiology. Sleep Med Clin.

[28] Tähkämö L., Partonen T., Pesonen A.K. (2019). Systematic review of light exposure impact on human circadian rhythm. Chronobiol Int.

[29] Touitou Y., Reinberg A., Touitou D. (2017). Association between light at night, melatonin secretion, sleep deprivation, and the internal clock: health impacts and mechanisms of circadian disruption. Life Sci.

[30] Dashti H.S., Gómez-Abellán P., Qian J. (2021). Late eating is associated with cardiometabolic risk traits, obesogenic behaviors, and impaired weight loss. Am J Clin Nutr.

[31] Zerón-Rugerio M.F., Longo-Silva G., Hernáez Á., Ortega-Regules A.E., Cambras T., Izquierdo-Pulido M. (2020). The elapsed time between dinner and the midpoint of sleep is associated with adiposity in young women. Nutrients.

